# Mechanism of Action and Clinical Efficacy of CDK4/6 Inhibitors in BRCA-Mutated, Estrogen Receptor-Positive Breast Cancers: Case Report and Literature Review

**DOI:** 10.3389/fonc.2019.00759

**Published:** 2019-08-13

**Authors:** Anna Maria Militello, Teresa Zielli, Daniela Boggiani, Maria Michiara, Nadia Naldi, Beatrice Bortesi, Paola Zanelli, Vera Uliana, Sara Giuliotti, Antonino Musolino

**Affiliations:** ^1^Breast Unit and Cancer Genetics Service, University Hospital of Parma, Parma, Italy; ^2^Medical Genetics Unit, University Hospital of Parma, Parma, Italy; ^3^Radiology Unit, University Hospital of Parma, Parma, Italy; ^4^Gruppo Oncologico Italiano di Ricerca Clinica (GOIRC), Parma, Italy

**Keywords:** BRCA, CDK4/6 inhibitor, breast cancer, estrogen receptor, cyclin D1, endocrine therapy, homologous recombination

## Abstract

Sensitivity to endocrine therapy of patients with estrogen receptor (ER)-positive metastatic breast cancer and germline *BRCA1/2* mutations is not yet fully elucidated. Furthermore, the registration trials of CDK 4/6 inhibitors in combination with endocrine therapy lacked of a pre-specified subgroup analysis in *BRCA1/2* mutation carriers. We report clinical history of two patients with BRCA-mutated, ER-positive metastatic breast cancer treated with letrozole plus the CDK 4/6 inhibitor palbociclib. Biological and clinical implications of the treatment outcome observed in the two cases are discussed with the knowledge of scientific evidence to date available. Overall, biological rationale, preclinical, and clinical data support the prominent role of CDK 4/6 inhibitors plus endocrine therapy, even in combination with PARP inhibitors, in the treatment of BRCA-mutated, ER-positive breast cancers. However, the interaction between Cyclin/CDK pathway, ER and BRCA is complex and evidences reported so far, albeit reliable, await confirmation in the context of future randomized clinical trials.

## Background

Up to 10% of breast cancers (BCs) are attributed to pathogenic germline mutations in the *BRCA1* and *BRCA2* genes (1, 2). Several studies on over 10,000 cases with BRCA-associated BCs have reported estrogen receptor (ER)-positive rates of 20 and 77%, in *BRCA*1 and *BRCA2* mutation carriers, respectively (3).

ER-positive BCs diagnosed in *BRCA1/2* mutation carriers are frequently characterized by higher tumor grade and proliferation rate than ER-positive tumors occurring in non-carriers (4). Less is known about sensitivity to endocrine therapy (ET) of patients with ER-positive metastatic BC (MBC) and *BRCA1/2* mutations (5, 6).

Three cyclin-dependent kinase (CDK) 4/6 inhibitors (palbociclib, ribociclib, and abemaciclib) have recently been approved for the treatment of ER-positive MBC (7). However, the registration trials of CDK 4/6 inhibitors in combination with ET lacked of a pre-specified subgroup analysis in *BRCA1/2* mutation carriers (8–10).

Here we report clinical history of two patients with BRCA-mutated, ER-positive MBC treated with letrozole plus palbociclib. Biological and clinical implications of the treatment outcome observed in the two cases are discussed with the knowledge of scientific evidence to date available.

## Case Presentation 1

In 2012, a 59 year-old woman underwent left upper paracentral quadrantectomy for pT2N1M0, stage IIB, G3, BC [ER: 40%, progesterone receptor (PR): 0%, HER2-negative, ki67: 40%]. The patient received adjuvant chemotherapy with FEC (epirubicin, cyclophosphamide, and 5 FU) for 3 cycles and Docetaxel for 3 cycles. Then, in May 2013, she started adjuvant radiotherapy and ET with anastrozole. Due to the presence of strong family history (FH) of breast and ovarian cancer, BRCA genetic testing was performed with detection of the c.5332+1G>A germline, pathogenic *BRCA1* mutation. The patient had prophylactic bilateral annessiectomy in October 2015.

In March 2016 the patient presented with sternal pain. Clinical examination revealed the presence of a chest-wall palpable mass. She had fine needle biopsy (FNB) of the sternal mass with cytological diagnosis of BC metastasis (ER: 60%, PR: 2%, HER2-negative). A total body computed tomography (CT) scan confirmed a unique metastatic bone lesion of the sternum. Anastrozole therapy was then stopped and on April 22, the patient started weekly chemotherapy with paclitaxel, which was interrupted after 2 months due to the CT evidence of an increase in size of the sternal lesion (longest diameter 67 vs. 57 mm). She was then enrolled in the experimental protocol “BRAVO” (11) and treated with the Poly(ADP-ribose) polymerase (PARP) inhibitor niraparib from August 2016 to February 2017, until the CT scan showed disease progression (best response: partial response [PR]). In view of the persistence of a single painful skeletal localization of disease, in March 2017 the patient underwent local radiotherapy and ET with fulvestrant was started concomitantly. After 2 months of therapy, the disease remained radiologically stable. In November 2017, the CT scan showed an increase of sternal lesion, and the CDK4/6 inhibitor, palbociclib, was added to ET. In June 2018, after 6 cycles of treatment, the CT scan showed stable disease (SD).

**Figure 1:**
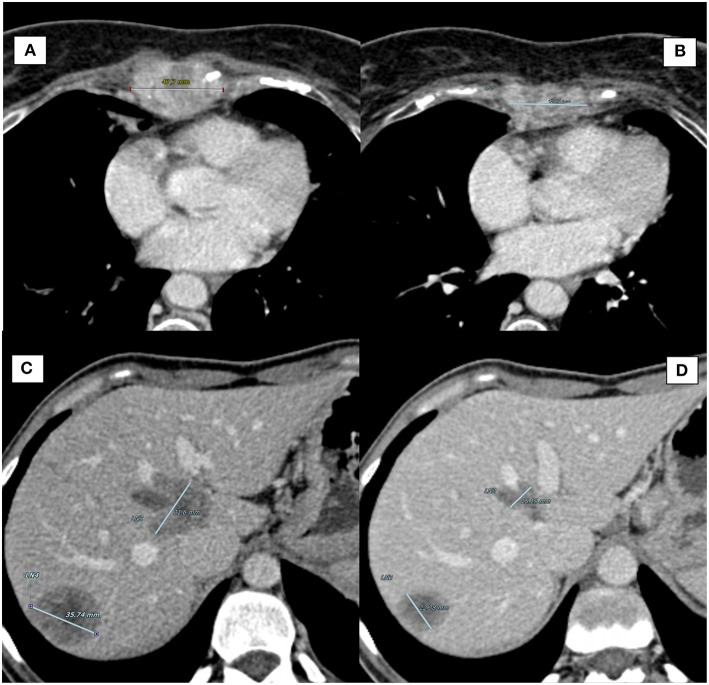
Computed Tomography (CT) scan of target lesions during therapy with CDK4/6 inhibitors. **(A)** Case report 1, sternal lesion at baseline (longest diameter: 47.7 mm). **(B)** Case report 1, sternal lesion at best response (longest diameter: 42.91 mm). **(C)** Case report 2, largest liver metastasis at baseline (longest diameter: 35.74 mm). **(D)** Case report 2, largest liver metastasis at best response (longest diameter: 22.38 mm).

The patient is currently in good clinical condition (ECOG PS 0, no sternal pain) and continuing treatment. The latest CT scan (10 January 2019) showed a gradual reduction of the sternal lesion [best response by RECIST criteria (12): SD; Figures 1A,B]. At the last visit (April 2019), the duration of response (DOR) is 10 months.

## Case Presentation 2

In August 2012, after self-detection of a left breast lump, a 32 year-old woman underwent fine biopsy of the breast nodule, which was positive for invasive ductal carcinoma. Due to the young age and the presence of a positive FH, BRCA genetic testing was performed with detection of the c.8878C>T germline pathogenic *BRCA2* mutation. In October 2012 the patient underwent both left and prophylactic right mastectomy. The left breast pathology report revealed the presence of a pT1cN1M0, stage IIB, G2, BC (ER: 100%, PR: 40%, HER2 negative, ki67: 25%).

From December 2012 to March 2013, the patient received adjuvant chemotherapy with FEC for 3 cycles followed by docetaxel for 3 cycles. Then, she started adjuvant endocrine therapy with LHRH analog in combination with tamoxifen.

In June 2016, a blood exam showed an increase of CA 15-3 along with CT scan documenting suspicious chest-wall lymph-nodes, liver, and bone lesions. FNB and cytology of lymph-nodes confirmed the presence of BC cells (ER: 100%, PR: 35%, HER2-negative). In July 2016, the patient started first line chemotherapy with capecitabine, with good PR. However, in April 2017, the CT scan showed disease progression in lung, liver, and thoracic lymph-nodes. The patient started second-line chemotherapy with eribulin, which was interrupted after 3 cycles due to pulmonary and liver progression. From August 2017 to February 2018 the patient received 9 cycles of Carboplatin (best response: PR). The treatment was then interrupted for further disease progression (neoplastic lymphangitis with dyspnea). In April 2018, the patient began a new treatment with palbociclib plus letrozole and LHRH analog. After 5 months of treatment, she reported resolution of dyspnea with PS improvement, and a significant response was observed by CT scan [best response by RECIST critera (12): PR; Figures 1C,D]. At the last visit (April 2019), the patient is currently on treatment with no evidence of disease progression (DOR: 7 months).

## Discussion

We reported clinical history of two patients with germline *BRCA1/2* mutations and refractory ER-positive MBC who achieved durable response to the combination of palbociclib and ET.

**Figure 2:**
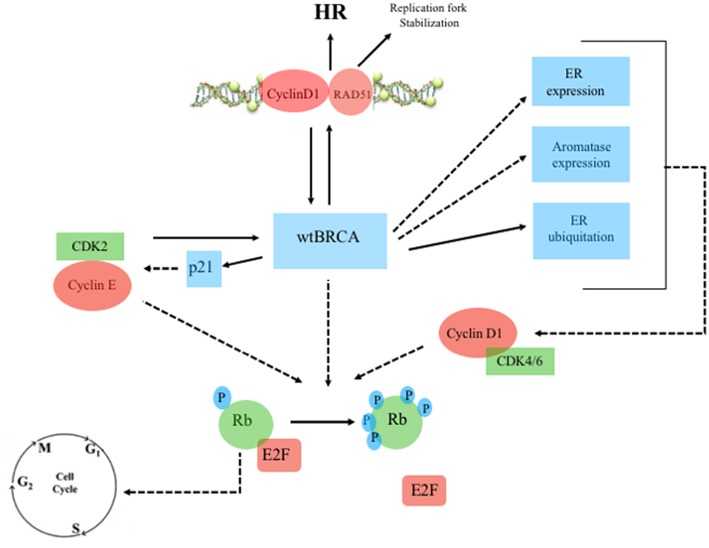
Wild-type BRCA, Estrogen Receptor and Cyclin/CDK pathway. Continuous line: activation, dashed line: inhibition. ERa, estrogen receptor-alpha; Rb, retinoblastoma; E2F, transcription factor; wtBRCA, wild-type BRCA; HR, homologous recombination; CDK, cyclin-dependent kinase; p21, endogenous CDK inhibitor; P, phosphorylation sites.

**Figure 3:**
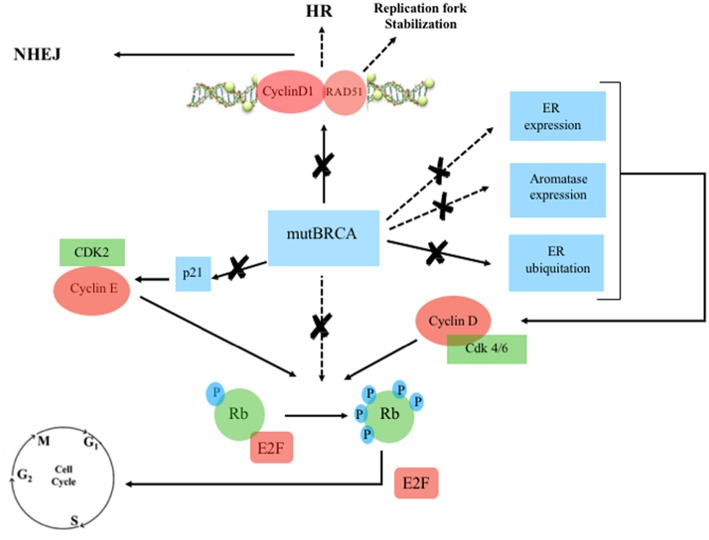
Mutated BRCA, Estrogen Receptor and Cyclin/CDK pathway. Continuous line: activation, dashed line: inhibition. ERa, estrogen receptor-alpha; Rb, retinoblastoma; E2F, transcription factor; mutBRCA, mutated BRCA; HR, homologous recombination; NHEJ, non-homologous end joining; CDK, cyclin-dependent kinase; p21, endogenous CDK inhibitor; P, phosphorylation sites.

**Figure 4:**
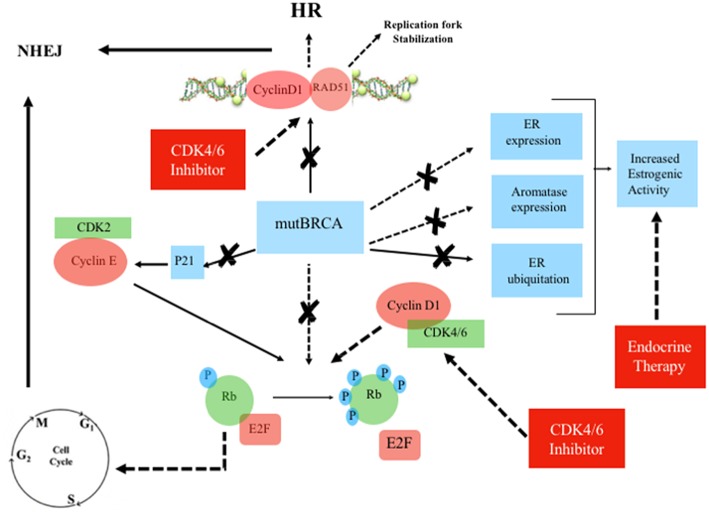
Mutated BRCA, Estrogen Receptor and Cyclin/CDK pathway in the presence of CDK4/6 inhibitor plus endocrine therapy. Continuous line: activation, dashed line: inhibition. ERa, estrogen receptor-alpha; Rb, retinoblastoma; E2F, transcription factor; mutBRCA, mutated BRCA; HR, homologous recombination; NHEJ, non-homologous end joining; CDK, cyclin-dependent kinase; p21, endogenous CDK inhibitor; P, phosphorylation sites.

There are multiple links between ER, BRCA, and CDK pathway (Figures 2, 3). Mote et al. suggested that altered expression of PR is a phenotype associated with mutations in *BRCA1* and *BRCA2* genes (13). Other *in vitro* studies suggested that the wild-type BRCA1 gene product interacts with and suppress the activity of ER-alpha (ESR1) through a direct physical and estrogen-independent interaction between the amino-terminal region of BRCA1 and the conserved carboxyl-terminal activation (AF-2) domain of ER-alpha (14). *BRCA1* deleterious mutations abolish or reduce BRCA1 ability to inhibit ER-alpha activity (14, 15). It is therefore suggested that BRCA1 could function as a brake on ER-alpha driven proliferation and *BRCA1* mutations can release that brake (16) (Figures 2, 3). Furthermore, Ma et al. showed that ER is also a target for the ubiquitin ligase activity of BRCA1 (17). Mutations of the ubiquitination sites abolish BRCA1-mediated inhibition of ER-alpha activity (17). BRCA1 is also able to inhibit P300-mediated ER acetylation, which is essential for the transactivation functions of ER (18). In addition to direct suppression of ER activity, wild-type BRCA1 also inhibits aromatase expression, thereby lowering estrogen levels and further reducing ER-alpha-mediated transcription (19). Overall, the higher activity of ER-alpha in BRCA mutation carriers could represent the rationale for a better sensitivity to ET in patients with ER-positive BC and *BRCA1/2* germinal mutations (Figure 4).

The overexpression of cyclin D1 is found in ~50% of BCs (20), while amplification of its corresponding *CCND1* gene has been observed in 9–30% of cases (21). Overexpression of cyclin D1 correlates with ER expression, PR expression, and luminal subtypes, with a favorable impact on overall survival (OS) in whole BC series containing all tumor phenotypes (21, 22). However, in the luminal A group, high expression of cyclin D1 has been associated with shorter disease-free survival (DFS), suggesting that the prognostic role of cyclin D1 depends on the molecular subtype (22). Patients with tumors with high amplification of *CCND1*, which are mostly of the luminal B subtype, have also been found been to have an increased risk of disease recurrence (21, 22). Cyclin D1 promotes the G 1/S-phase transition by binding and activating CDK4 and CDK6 (23), and CDK4/6 inhibitors effectively block the proliferation of sensitive cancer cells by inducing G1 cell cycle arrest (24). Cyclin D1 has also CDK-independent functions, such as ligand-dependent inhibition of the Androgen Receptor (AR) (25), and the promotion of transcription of the ER-alpha gene (26). Furthermore, Cyclin D1 stabilizes the interaction between ER and SRC-1 via direct binding to both proteins (27). ER also regulates the expression of the gene encoding cyclin D1 (28). There is therefore a positive feedback loop: activation of cyclin D1 leads to ER expression, which induces more cyclin D1 production (29). Wild-type BRCA is also involved in G1 cell cycle arrest. Aprelikova et al. showed that BRCA1 binds to hypophosphorylated RB, which interacts with the E2F transcription factor to block transcription and inhibit cell proliferation (30). In the presence of *BRCA1* mutations, this antiproliferative control fails. However, in those cases, CDK4/6 inhibitors may restore the G1 arrest, preventing the cell from entering mitosis (31) (Figure 4). Somasundaram et al. also suggested that BRCA1 contributes to cell cycle arrest and tumor growth suppression through the induction of the CDK2 inhibitor p21 (32) (Figures 2, 3). CDK2 is a key bypass kinase of CDK4/6 inhibition and high mRNA expression of cyclin E1 (*CCNE1*), which activates CDK2, has been associated with resistance to palbociclib in the PALOMA-3 trial (33). It is interesting to note that, although the loss of BRCA1 may induce CDK2 activity through p21 inhibition, on the other hand, *CCNE1* amplification and cyclin E1 protein overexpression have been reported to be mutually exclusive with *BRCA1/2* mutations (34, 35).

Cyclin D1 also plays a cell-cycle-independent role in DNA repair. In response to DNA double-strand breaks (DSBs), it recruits proteins involved in the homologous recombination (HR) DSB repair pathway such as BRCA1 (36), RAD 51 (37), and BRCA2 (38). Interestingly, the recruitment itself of cyclin D1 to sites of DNA damage has been shown to be BRCA2-dependent (38) (Figures 2, 3). It is important to note that HR provides accurate recombination using a sister chromatid as a template, maintaining genomic stability. However, due to the need for a sister chromatid, HR is limited to the S-phase and G2-phase of cell cycle (39). By using CDK4/6 inhibitors, cells are arrested in G1 phase where they do not have an available sister chromatid in case of DNA damage and are dependent upon the compensatory, error-prone, non-homologous end joining (NHEJ) pathway, which is less accurate and may thus promote mutations and genomic instability (40). Furthermore, a decrease of HR activity during DNA damage may be induced by CDK4/6 inhibitors also in a cycle independent manner, due to the repression of critical factors for HR such as RAD51 (41). Therefore, in patients with HR deficiency (HRD), such as BRCA mutation carriers, who are treated with CDK 4/6 inhibitors, any DNA damage may cause cancer cell death since CDK4/6 inhibitors induce compensatory activation of the less accurate NHEJ pathway and impair recruitment of RAD51 (41) (Figure 4). These findings suggest that CDK inhibitors may exert synthetic lethal effect against BRCA-mutated, ER-positive BCs (37, 38).

PARP is constituted by several enzymes, which facilitate DNA repair in pathways involving single-strand breaks (SSBs) and base excision repair (BER) (42). In the presence of a PARP inhibitor, attempted DNA repair of SSB results in DSB formation. BRCA-proficient cells have the ability to repair the DSB, maintaining survival, but BRCA-deficient cells are unable to repair the accumulating DSBs which lead to cell synthetic lethality (42). The randomized, phase 3 OlympiAD and EMBRACA trials showed that, among patients with HER2-negative MBC and germline BRCA mutation, PARP inhibitors olaparib and talazoparib provided a significant progression-free survival (PFS) benefit over standard therapy (43, 44). Interestingly, subgroup analysis in the OlympiAD trial showed a greater efficacy of olaparib in patients with triple-negative breast cancer (TNBC) than in ER-positive ones, and also a better outcome in *BRCA1* than in *BRCA2* mutation carriers (43). However, in the final analysis of the study, OS, a secondary end point, was not significantly different between treatment arms both in the whole study population and among the predefined subgroups (45). At present, several ongoing trials are evaluating the combination of CDK inhibitors with PARP inhibitors (46). The combination of dinaciclib (a CDK 1, 2, 5, 9, and 12 inhibitor) with the PARP inhibitor veliparib has shown preliminary clinical benefit in patients with breast cancer, prostate cancer, ovarian cancer, and other gynecologic malignancies (47). By blocking CDK12, CDK1, or CDK2, BRCA wild-type cancer cells turn into HR-deficient cells through inhibition of BRCA1 expression and phosphorylation (48, 49) and, thus, become more susceptible to synthetic lethality induced by PARP inhibitors (46–49). A phase I/II trial of olaparib, palbociclib, and fulvestrant has been recently activated in BRCA-mutated patients with ER-positive, HER2-negative MBC^1^.

## Conclusion

The Cyclin/CDK pathway has a crucial role in the regulation of cell cycle progression. In the last years, CDK4/6 inhibitors have changed the treatment landscape for ER-positive BC. The clinical reports here presented show a significant activity of CDK4/6 inhibitors in BRCA-mutated, ER-positive BC patients.

BRCA genes are involved in the regulation of DNA Repair Mechanism, but are also strategic for ER expression and function. In the presence of BRCA mutations, which abolish or reduce BRCA-dependent ER-alpha inhibition, the use of ET could restore the brake on ER-alpha driven proliferation. Moreover, due to BRCA mutations, cell cycle arrest fails, but CDK4/6 inhibitors could restore G1 arrest. The induction of G1 cell cycle arrest can be used to manipulate the activity of DNA repair pathways, especially in HR-deficient cells. In those cells, G1 cell cycle arrest may lead to a major activity of NHEJ, with consequent genomic instability and apoptosis.^1^

Overall, biological rationale, preclinical, and clinical data support the prominent role of CDK 4/6 inhibitors plus endocrine therapy, even in combination with PARP inhibitors, in the treatment of BRCA-mutated, ER-positive breast cancers. However, the interaction between Cyclin/CDK pathway, ER and BRCA is complex and evidences reported so far, albeit reliable, await confirmation in the context of future randomized clinical trials.

## Data Availability

All datasets generated for this study are included in the manuscript and/or the supplementary files.

## Ethics Statement

The authors of this manuscript obtained patient consent for publication of clinical data and images. The patients' details were anonymized and signed consent forms were attached to the medical records. Due to the retrospective and non-interventional nature of the study, permission by the local ethics committee was not required.

## Author Contributions

AMi and TZ contributed equally to the writing of the manuscript and designed the figures. AMu and DB were involved in planning and supervised the study. SG interpreted the radiological images. MM, NN, BB, PZ, and VU reviewed and approved the final version of this work.

### Conflict of Interest Statement

The authors declare that the research was conducted in the absence of any commercial or financial relationships that could be construed as a potential conflict of interest.

## Footnotes

^1^Olaparib, Palbociclib, and Fulvestrant in Patients With BRCA Mutation-associated, Hormone receptor-positive, HER2-negative metastatic Breast Cancer – NCT03685331. Available online at: https://clinicaltrials.gov/ct2/show/NCT03685331.
